# Sudden Sensorineural Hearing Loss in Mild Covid-19: Case Series and Analysis of the Literature

**DOI:** 10.3390/audiolres11030029

**Published:** 2021-07-01

**Authors:** Filippo Ricciardiello, Davide Pisani, Pasquale Viola, Elisabetta Cristiano, Alfonso Scarpa, Antonio Giannone, Giuseppe Longo, Giuseppe Russo, Marco Bocchetti, Ciro Coppola, Marco Perrella, Flavia Oliva, Giuseppe Chiarella

**Affiliations:** 1 ENT Department, AORN Cardarelli, 80131 Napoli, Italy; filipporicciardiello@virgilio.it (F.R.); cristianoelisabetta@outlook.it (E.C.); antonio.giannone@aocardarellli.it (A.G.); flaviaoliva311@gmail.com (F.O.); 2 Unit of Audiology, Department of Experimental and Clinical Medicine, Magna Graecia University, 88100 Catanzaro, Italy; pasqualeviola@unicz.it (P.V.); chiarella@unicz.it (G.C.); 3 Department of Medicine and Surgery, University of Salerno, 84084 Fisciano, Italy; alfonsoscarpa@yahoo.it; 4 AORN Cardarelli, 80131 Napoli, Italy; glongo@gmail.com; 5 Health Management AORN Cardarelli, 80131 Napoli, Italy; ariete_gr@libero.it (G.R.); ciro.coppola@aocardarelli.it (C.C.); 6 Biogem Scarl, Molecular Oncology and Precision Medicine Laboratory, 83031 Irpino, Italy; marco.bocchetti@unicampania.it; 7 Anesthesiology and Reanimation Department AORN Cardarelli, 80100 Napoli, Italy; Marco.Perrel@gmail.com

**Keywords:** coronavirus, SARS-CoV-2, COVID-19, sudden sensorineural hearing loss, SSNHL, tinnitus, vertigo, hearing loss, treatment

## Abstract

Background: There is growing evidence of otoneurological involvement of SARS-CoV-2, such as tinnitus and balance disorders and smell and taste disorders, but HL in COVID-19 patients has still been marginally studied. Investigating the role of SARS-CoV-2 as an aetiological factor of Sudden Sensorineural Hearing Loss (SSNHL) may offer the opportunity to address treatment strategies to maximize clinical recovery and avoid side effects. Methods and results: For this purpose, we will present case studies of five patients who experienced SSNHL during COVID-19. Patients were selected from COVID-19 positive adult subjects with mild clinical presentation, admitted to the outpatient Ear Nose and Throat Department of Cardarelli Hospital due to the onset of SSNHL during the infection. All underwent a complete audio-vestibular investigation before and after SSNHL treatment protocol. Each patient is described with a detailed analysis. Conclusions: SSNHL could be an occasional symptom of COVID-19, even in mild manifestations of the disease. Our experience leads us to underline the value of promptly recognizing and addressing this and other uncommon symptoms, giving patients the opportunity to receive early treatment.

## 1. Introduction

Sudden Sensorineural Hearing Loss (SSNHL) is defined as a hearing loss (HL) greater than 30 dB, over at least 3 contiguous audiometric frequencies, occurring suddenly or within 3 days. The cause of SSNHL remains unknown in most patients. Although several aetiological hypotheses have been proposed, the most likely causative sources seem to be cochlear injury from impaired vascular perfusion or viral infection [1].

Idiopathic SSNHL spontaneously recovers within two weeks in 32–65% of cases [2]. The observation that SSNHL has an acute onset, is generally unilateral and can resolve within a few hours or days suggests that inner ear microcirculation disturbances can play a key role in this syndrome. Disorders of cochlear blood flow due to alteration in plasma viscosity, cellular and platelet aggregability, red blood cell deformability and endothelial function have been reported in patients affected by SSNHL [3].

This syndrome has a prevalence of one in 10,000–15,000 persons and about half of them have concurrent vertigo. It mostly afflicts people between 40 and 54 years old [4,5]. Vertigo may be present at the beginning of the SSNHL or may appear later. Vestibular impairment is highly associated with severe HL and should be addressed as an independent, negative, prognostic factor [6]. Prompt recognition of SSNHL is crucial and early medical intervention could maximize treatment effectiveness [7].

Neurological manifestations of viral infections are well known [8,9,10]. In SSNHL, the viral action can directly damage cochlear nerves and/or cochlear structures or induce responses resulting from the cross-reaction of the inner ear antigens [11]. Animal studies have reported viral induction of HL due to direct inner ear involvement or indirectly via cerebrospinal fluid [12,13,14,15,16].

During the previous SARS epidemic, coronavirus infections were reported to be associated with loss of smell and taste due to neural injury [17,18]. Intranasal delivery of SARS-CoV-2 has shown neuroinvasion and encephalitis in mice [19].

Even if there is growing evidence of otoneurological involvement of SARS-CoV-2, such as tinnitus and balance disorders [20,21] and smell and taste disorders [22,23,24], HL in COVID-19 patients has still been marginally studied.

Investigating the role of SARS-CoV-2 as an aetiological factor of SSNHL may offer the opportunity to address treatment strategies to maximize clinical recovery and avoid side effects. For this purpose, we will present in this study case studies of patients who experienced SSNHL during COVID-19.

## 2. Materials and Methods

This study was carried out from September to December 2020, at Cardarelli Hospital of Naples, Italy. Patients were selected from COVID-19 positive adult subjects, admitted to the outpatient Ear Nose and Throat (ENT) Department, due to the onset of SSNHL during infection.

The inclusion criteria were:

- age between 18 and 65 years;
- diagnosis of SARS-CoV-2 infection, through a polymerase chain reaction (PCR) assay on oropharyngeal and nasopharyngeal (OP/NP) swab test [25];
- asymptomatic to mild clinical presentation of COVID-19, according to NIH Guidelines [26];
- compliance with the therapy and adherence to the follow-up.

None of our patients had:

- previous HL and/or tinnitus;
- previous ear pathology and/or otologic surgery;
- cerebello-pontine angle pathology or congenital ear malformations;
- use of ototoxic drugs [27,28];
- head and/or neck trauma or barotrauma within the last 3 months.

Five patients were enrolled in this study. Informed consent was obtained from all subjects. All underwent a clinical audio-vestibular investigation, including micro-otoscopy, pure tone audiometry (PTA), acoustic immittance test, auditory brainstem evoked response (ABR), acufenometry, bedside vestibular examination and video head impulse test (v-HIT).

Self-assessment questionnaires regarding tinnitus (THI—Tinnitus Handicap Inventory) [29] and dizziness (DHI—Dizziness Handicap Inventory) [30] were administered before and after treatment.

PTA (measured at 125, 250, 500, 1000, 2000, 3000, 4000, and 8000 Hz) and acufenometry (loudness e pitch measures) were performed using Piano Clinical Audiometer (Inventis, Padua, Italy) inside a soundproof audiometric booth; acoustic immittance measurements were performed using AT 235 Tympanometer (Interacoustics, Denmark).

The otoneurological-ABR procedure was performed at 0.1 ms click stimulus, alternated polarity, 21 pps rate with 2000 repetitions and acquisition interval of 10 ms with HP of 100 Hz and LP of 3000 Hz (ICS Chartr EP 200, Otometrics, Taastrup, Denmark).

The v-HIT was performed using ICS-impulse^®^ equipment (GN Otometrics, Taastrup, Denmark).

Before starting the first cycle of SSNHL treatment, each patient underwent a blood screen test, including complete Blood Count (CBC), Mean Platelet Volume (MPV), Erythrocyte Sedimentation Rate (ESR), C-Reactive Protein (CRP), cholesterol assessment (total, HDL, LDL), triglycerides, homocysteine, blood glucose and fibrinogen, International Normalised Ratio (INR), Neutrophil-to-Lymphocyte Ratio (NLR), Platelet-to-Lymphocyte Ratio (PLR) and TORCH IgM and IgG screen tests.

Audiological and vestibular tests were performed before each treatment.

All patients received first-line COVID-19 medical treatment: Azithromycin (500 mg once a day for three days) and Paracetamol (1000 mg up to three times a day). Glucocorticoids were used for a short time (3–5 days) and the dose was not more than the equivalent of 1–2 mg/kg methylprednisolone per day. All drugs were orally administered.

Once they had been referred to our ENT Department for the sudden onset of SSNHL, all patients received oral high dose prednisone therapy (60 mg/day) [31,32,33], oral mesoglycan (50 mg, twice a day) [34,35] and Hyperbaric Oxygen Therapy (HBOT) [36,37,38,39]. During HBOT, 100% oxygen was provided at a pressure of 2.5 ATA, in sessions of 1 h per day. Each cycle of therapy lasted seven days. Since the Oxygen Chamber was barred to SARS-CoV-2 positive patients, a swab test was performed before each cycle of therapy. If the patients had risk factors for or a history of cardiovascular diseases, low-molecular-weight heparin s.c. (LMWH) was used (4000 units of Enoxaparin twice a day). We decided to treat residual dizziness with a cycle of vestibular rehabilitation.

We used modified Siegel’s criteria to assess the grade of recovery after treatment [40]:

- complete recovery (CR): final hearing better than 25 dB;
- partial recovery (PR): more than 15 dB gain, final hearing 25–45 dB;
- slight improvement (SI): more than 15 dB gain, final hearing poorer than 45 dB;
- no improvement (NO): less than 15 dB gain, final hearing poorer than 75 dB.

All patients underwent a brain CT and MRI assessment, which excluded brain diseases in all of them.

All patients started the first cycle of treatment the day after the audiological assessment. They underwent all prescribed cycles of treatment and then were referred to a long-term follow-up protocol.

Data were analyzed using Statistical Packages for Social Sciences (SPSS), version 26 (SPSS Inc., Chicago, IL, USA). The paired samples *t*-test was used to compare numerical values before and after treatment.

## 3. Results—Case Series

### 3.1. Patient #1

Healthy female, 26 years old, without history of disease, who tested positive for SARS-CoV-2 on 12 October 2020 while her only symptom was a slight asthenia. She did not need any medical therapy; on 20 October, she reported tinnitus and fullness in her left ear. A new OP/NP swab tested negative for SARS-CoV-2 on 22 October. The same day she complained of vertigo, hyperacusis, an increased tinnitus intensity and perception of almost total HL in the left ear. The next day, she underwent a complete audiological assessment: PTA showed normal hearing threshold in the right ear and deep-severe left ear SSNHL, with tonal field limitations at acute frequencies (3–8 kHz), and acufenometry revealed a 6 dBSL, continuous, 4 kHz tinnitus. The THI scored 60 points (4th degree).

The vestibular examination revealed a left static-dynamic hypofunction, with a 3rd degree, right-sided, spontaneous nystagmus (Ny) and almost totally deficient left Vestibulo-Ocular Reflex (VOR); the Head Shaking Test (HST) generated a right-sided Ny, while the clinical Head Impulse Test (c-HIT) test was left-positive.

The v-HIT showed a left gain deficit. The DHI test scored 82 (severe handicap). The ABR was normal on the right side, while it was not evocable on the left side. Blood testing revealed Toxo-IgG, ESR and PCR were slightly increased.

She underwent the first two cycles of treatment with prednisone, mesoglycan and HBOT.

After each treatment, PTA showed a 10 dB auditory recovery at low frequencies (125-250-500 Hz), the spontaneous nystagmus became 2nd degree and the results of other tests were unchanged compared to the previous assessment.

Prednisone was suspended; the patient continued mesoglycan therapy and underwent a third cycle of HBOT. She reported improved left auditory perception, while imbalance and tinnitus did not change. On 13 November PTA showed a further 10 dB recovery at low frequencies, while vestibular examination did not show changes. All drug therapy was suspended, and the patient started a vestibular rehabilitation cycle for her residual dizziness.

During the next audiological assessment, on 23 November, the patient reported improved left auditory perception and less imbalance. Tinnitus improved to 4 dBSL, while PTA showed a further 10 dB auditory recovery at low frequencies and the appearance of a 120 dB auditory threshold at 3–4 kHz.

**Figure 1:**
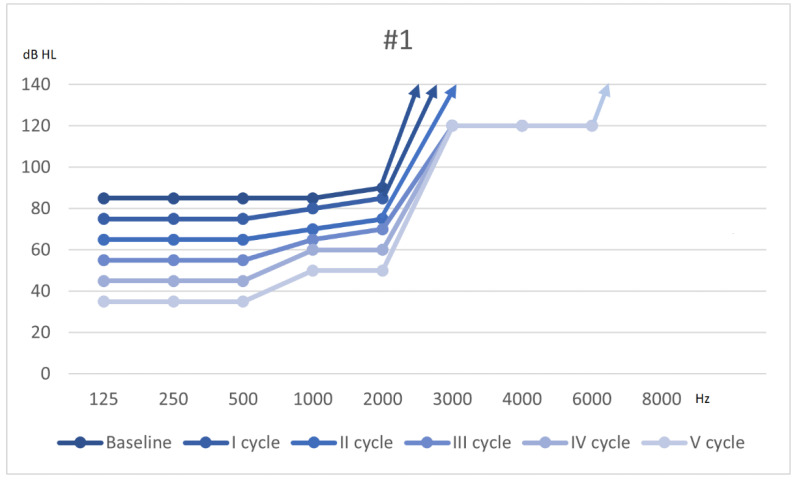
Hearing threshold at baseline, after 3 cycles of therapy and during the vestibular rehabilitation (IV and V).

On 30 November, PTA revealed an additional 10 dB recovery at low frequencies and the appearance of a 6 kHz auditory threshold at 120 dB. Tinnitus changed slightly compared to the previous assessment; acufenometry revealed a 3 dBSL continuous 4 kHz tinnitus, while the THI test scored 32 (grade 2). Bedside vestibular examination did not show spontaneous nystagmus, and the HST generated a direct right sided Ny. The patient continued vestibular rehabilitation, which resulted in a clear improvement of the dizziness. One month later her DHI score was 28. She received a diagnosis of slight to moderate SNHL at low-mid frequencies and profound SNHL at acute frequencies. The patient had a clear improvement of her hearing threshold as shown in Figure 1; following Siegel’s criteria she had a partial recovery. The patient was referred to a long-term follow-up protocol and is still working on her residual dizziness.

### 3.2. Patient #2

Healthy male, 22 years old, without history of disease. He complained about loss of smell and taste as only symptoms; his OP/NP swab tested positive for SARS-CoV-2 on 27 September 2020. A first cycle of medical therapy with azithromycin and prednisone was performed, with complete recovery of taste and smell.

On October 3rd, he complained of a sudden onset of dizziness, right ear fullness and HL with a “metallic” perception of sounds. PTA showed a normal threshold in the left ear and a severe degree (60 dB) right SNHL at low frequencies (125-250-500 Hz), rising at mid-high frequencies (1000-2000-3000-4000 Hz, 55-50-35-35 dB respectively). The 6 kHz and 8 kHz frequencies had normal thresholds. The patient did not report vertigo and tinnitus, and complied with THI and DHI tests, which scored 0. The patient received therapy with prednisone and mesoglycan.

On 6 October, his OP/NP swab tested negative for SARS-CoV-2 and he reported an improved auditory perception and the “metallic” perception disappeared. Drug therapy continued and a first cycle of HBOT was prescribed.

On 10 October PTA showed a 5 dB improvement at low frequencies, while the 2 kHz frequency had a 25 dB improvement. The 3–4 kHz frequencies showed a normal threshold. He started a second cycle of prednisone and mesoglycan therapy, while continuing the ongoing first cycle of HBOT, after which a new cycle of HBOT was prescribed.

On 17 October PTA revealed an improved 2000 Hz frequency threshold, which was within normal range, while moderate SNHL persisted at low frequencies (average threshold of 50 dB) and mild SNHL at 1000 Hz (35 dB). The ABR was normally evocable, and the bedside vestibular examination was normal. The v-HIT showed a right gain deficit. The DHI score was 0.

A third cycle of prednisone and mesoglycan was prescribed, while the ongoing second HBOT cycle continued.

On 24 October, audiometric examination showed a 10 dB improved low frequencies threshold while the 1000 Hz frequency was within normal parameters. The vestibular examination was normal. He complied with THI and DHI tests, which scored 0 points.

**Figure 2:**
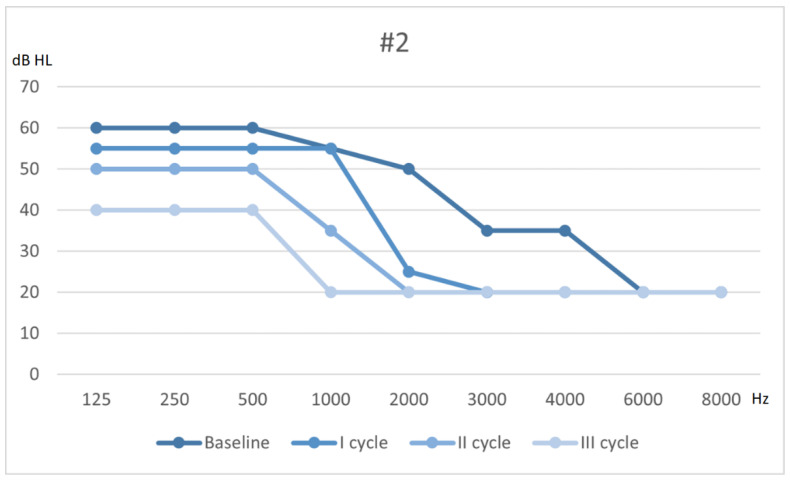
Hearing threshold from baseline to outcome (after 3 cycles of therapy).

The patient received a diagnosis of slight SNHL in the right ear and normoacusis in the left ear. His recovery was defined as partial, following Siegel’s criteria. Figure 2 shows his hearing threshold improving after each cycle of therapy. He was referred to a long-term follow-up protocol.

### 3.3. Patient #3

A 61-year-old male, with hyposmia, hypogeusia, fever and severe headache, who tested positive for SARS-CoV-2 on 3 September 2020.

Due to his high cardiovascular risk (previous acute coronary syndrome, hypertension, and diabetes) he received 4000 IU of Enoxaparin twice a day as well as azithromycin and prednisone. On 15 September he complained about a sensation of HL and tinnitus in his left ear, without vestibular symptoms, while previous symptoms improved significantly by 17 September. The patient underwent a new OP/NP swab test on 21 September, which was negative.

The same day he underwent a complete audiological assessment. PTA showed a normal right ear threshold and profound left SNHL (95 dB HL threshold) with an acute frequencies tonal field limitation (3–8 kHz). Acufenometry showed a continuous 3 kHz sound of 6 dB SL intensity, while the THI test scored 64 points (grade 4). The bedside vestibular examination and v-HIT were normal, DHI scored 0.

ABR was normal on the right side while the left side had no responses.

The blood test revealed HSV-IgG and high levels of homocysteine.

He received a first cycle of medical therapy with prednisone, mesoglycan and HBOT.

On 5 October, PTA showed a 15 dB HL improvement at low and mid frequencies (125–2000 Hz) and the appearance of a 105 dB HL auditory threshold at high frequencies (3–8 kHz). The vestibular examination was normal.

Prednisone therapy was suspended due to a worsening of his glycaemic homeostasis; mesoglycan therapy and a second HBOT cycle were completed.

On 12 October, PTA showed an overall improvement: he was diagnosed with a severe flat-shaped SNHL with a threshold of 70–75 dB HL.

**Figure 3:**
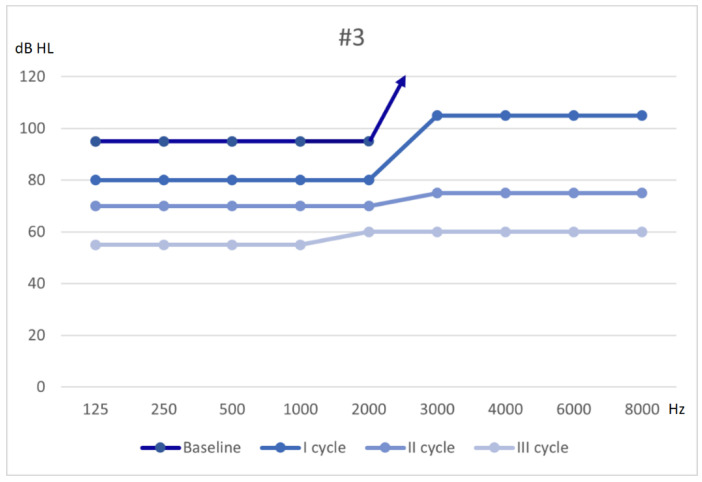
Hearing threshold from baseline to outcome (3 cycles of therapy).

A third cycle of HBOT and mesoglycan was administered, and PTA showed an overall further improvement with a moderate-severe SNHL (flat 55–60 dB threshold); acufenometry showed a 2 dB SL decrease, THI scored 36 (grade 2) and DHI scored 0. His recovery was defined as slight, following Siegel’s criteria, and hearing thresholds are shown in Figure 3. The patient was referred to a long-term follow-up protocol.

### 3.4. Patient #4

Healthy male, 30 years old, without history of disease, who complained about asthenia on 14 October 2020; two days later he had fever and breathing difficulties. The OP/NP swab test was positive for SARS-CoV-2 and he underwent drug therapy with azithromycin and prednisone. COVID-19 had a course of about 20 days, with headache and asthenia as the only symptoms.

On 22 October, he complained about the sudden onset of intense left ear tinnitus and HL. Tinnitus and HL improved significantly after medical therapy.

A second OP/NP swab test resulted negative for SARS-CoV-2 on 2 November.

On 4 November, the patient reported left ear fullness as the only symptom. The same day he underwent a complete audiological assessment. PTA revealed a mild degree (35–40 dB) of right SNHL, and a severe degree (70–75 dB) of left SNHL, both at high frequencies (4–8 kHz). The ABR was normal. Acufenometry revealed a 4 kHz 3 dB SL tinnitus, and THI scored 23 (grade 2).

The bedside and v-HIT vestibular examinations were normal and the DHI score was 0.

Prednisone and mesoglycan drug therapy were prescribed, as well as a first cycle of HBOT.

The blood test results were normal.

After the first cycle of therapy, PTA was slightly improved (5–10 dB HL) on both sides, except for the 3 kHz frequency of the left ear, which had a 20 dB HL improvement. The vestibular examination and v-HIT did not report any change compared to the previous one.

The patient underwent a second cycle of HBOT and mesoglycan, while prednisone therapy was suspended. After the second cycle of treatment, PTA showed an effective improvement in both ears. He fully recovered his right-side hearing, and he was diagnosed with a slight-moderate HL in the left ear.

**Figure 4:**
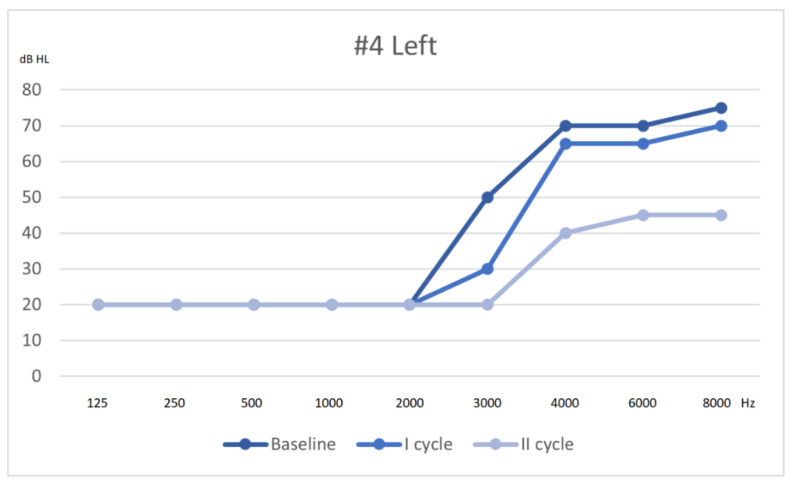
Hearing threshold of the left ear, from baseline to outcome (after 2 cycles of therapy).

**Figure 5:**
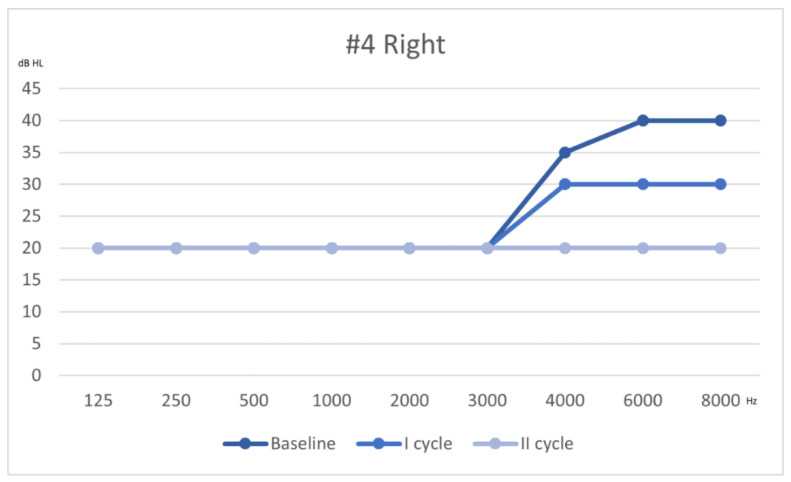
Hearing threshold of the right ear, from baseline to outcome (after 2 cycles of therapy).

Following Siegel’s criteria, he had a complete recovery on the right ear, partial on the left ear. The hearing thresholds from first assessment to outcome are shown in Figure 4 and Figure 5.

Acufenometry showed a 1 dB SL tinnitus, while the THI test scored 12 (grade 1). The DHI score was 0.

He was referred to a long-term follow-up protocol.

### 3.5. Patient #5

Healthy 46-year-old woman, without history of disease, whose OP/NP swab tested positive for SARS-CoV-2 on 24 November 2020; three days later she felt asthenia, joint pain, fever and headache. She underwent a first cycle of drug therapy with azithromycin and prednisone. On 30 November, she complained about bilateral tinnitus and right ear fullness. Tinnitus (worse in the right ear), and fullness persisted during the treatment, while she recovered from other symptoms.

A further OP/NP swab tested negative on 9 December. The same day she underwent a complete audiological assessment. PTA revealed moderate SNHL at 125-250-500 Hz (average 50 dB HL) and mid frequencies (35 dB HL at 1000 Hz). Acufenometry showed a continuous right-sided 750 Hz 5 dB SL tinnitus; the THI scored 42 (grade 3). She did not complain about vertigo or dizziness; the DHI scored 0.

The ABR was normal. The bedside vestibular and v-HIT examinations were normal.

**Figure 6:**
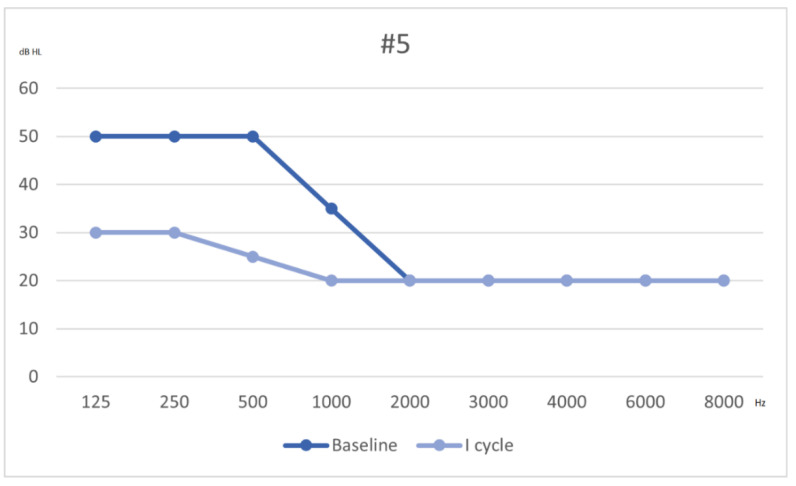
Hearing threshold from baseline to outcome (after 1 cycle of therapy).

She underwent a cycle of prednisone, mesoglycan and HBOT therapy. After this first cycle of treatment, she almost completely recovered: PTA showed normal thresholds, except for 250 Hz and 500 Hz, with 30 and 25 dB, respectively. The tinnitus disappeared, thus acufenometry was not performed and THI scored 0; DHI again scored 0. Following Siegel’s criteria, she had a partial recovery (Figure 6). The patient received a diagnosis of slight low frequency HL and was referred to a follow-up protocol.

## 4. Discussion

During the previous SARS and MERS epidemics, autoptic findings of viral nucleic acid in the cerebrospinal fluid were reported in patients with neurological involvement [41,42]. The presence of SARS-CoV-2 in the Central Nervous System (CNS) has been repeatedly reported, similarly to previous coronavirus outbreaks, in autoptic results of patients with SARS-CoV-2 that showed hyperemic and edematous brain tissue with neuronal degeneration [43,44]. Coronaviruses can penetrate the CNS and cause brain infections, even without signs and symptoms of neurological involvement [45]. Animal models have shown that the dispersal pathways through the CNS are the circulatory system, axonal transport, trans-neuronal spread and damaged areas of the blood–brain barrier and/or blood–nerve barrier [46].

Despite the small number of studies, the possibility of a relationship between COVID-19 and SSNHL is recently gaining prominence in the scientific literature. Indeed, a review of the existing literature revealed eight reports including 13 cases of SSNHL during the course of COVID-19 [47,48,49,50,51,52,53,54]. Most of them involved patients with critical COVID-19 presentations [47,48,49,50,51].

Chern et al. described a bilateral moderate-severe SSNHL in an 18-year-old patient with intralabyrinthine bilateral haemorrhage [52]. This case received a SARS-CoV-2 diagnosis via serum IgG, several weeks after the onset of audiological symptoms (fullness, vertigo, HL), and this makes it more difficult to properly address the SSNHL as a COVID-19 related event, and to compare it with our case series.

Kilic et al. reported five cases of SSNHL associated with COVID-19, who received therapy of high dose prednisolone and hydroxychloroquine until COVID-19 was resolved [53]. This could be the most representative study, with an excellent description of the pathophysiological mechanisms, but the patients’ hearing status was obtained only through phone interviews and the first audiological assessment took place at least one month after treatment. Moreover, these patients were exposed to hydroxychloroquine, a well-known ototoxic drug.

We found the only comparable report in a case of a 30-year-old female, who had a unilateral SSNHL during COVID-19 and received medical treatment with high dose steroids and a proper audiological assessment, without improvement [54].

To date, our study presents the largest cohort of patients affected by sudden audio-vestibular symptoms during COVID-19, providing a timely assessment and a strict follow-up. All our patients received a prompt imaging evaluation and a complete blood screen to exclude other causes of audio-vestibular symptoms and strengthen the hypothesis of a direct connection with COVID-19.

All our patients had normal NLR and PLR values, which are considered risk factors for SSNHL and predictors of poor prognosis [55,56].

We enrolled only patients with asymptomatic to mild COVID-19 clinical presentation, to avoid misinterpretation of clinical data that could be due to the severity of the syndrome. Our data suggest that SSNHL represents a manifestation of COVID-19, even in milder presentations of the disease. Therefore, SSNHL could be unrelated to clinical COVID-19 severity and could be experienced even during the healing phase of the infection.

**Figure 7:**
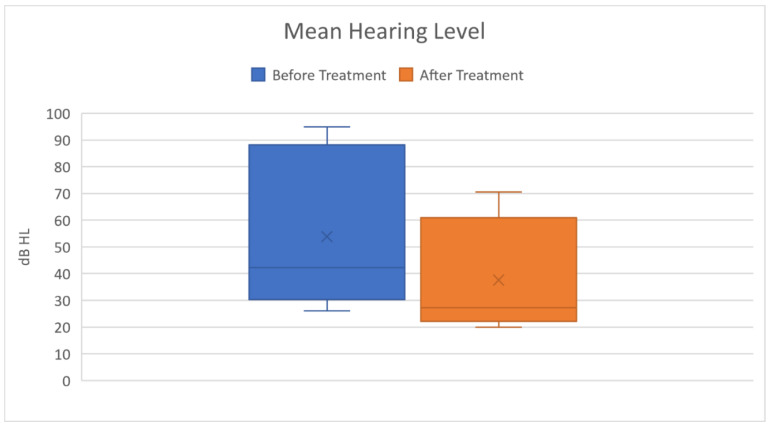
The overall Mean Hearing Level had a significative improvement after treatment (*p* = 0.0016).

**Figure 8:**
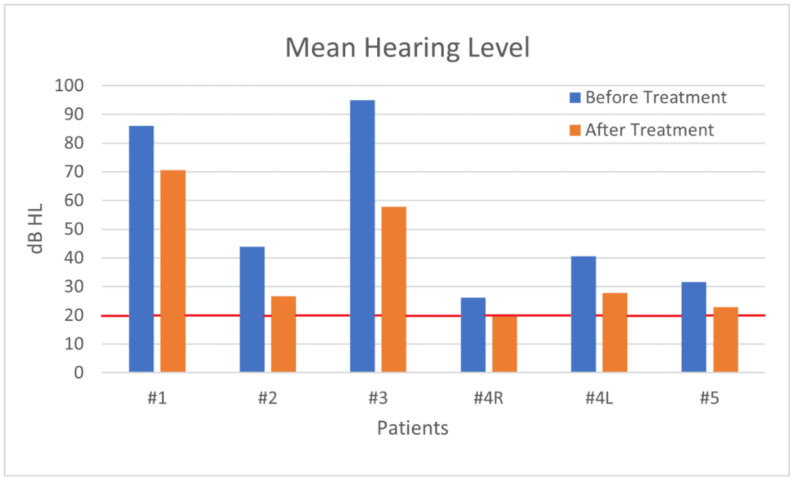
Each patient had an improved mean hearing level after treatment.

In our study, no patient experienced SSNHL as a first symptom of COVID-19; they revealed audio-vestibular symptoms >6 days after SARS-CoV = 2 diagnosis (mean 8.4 days). Patient no.1 experienced SSNHL after her recovery from SARS-CoV2 infection. Patients no.1 and no.3 had left HL, patients no.2 and no.5 had right HL, and patient no. 4 had bilateral SSNHL. As far as we know, this is the second case described in literature of young adults. Most of our patients achieved partial recovery of auditory function, with patient No.4 having a complete recovery on the right ear. Patient no.3 had only a slight recovery, probably due to his comorbidities. The overall (Figure 7) and individual patients’ (Figure 8) mean hearing level graphical analyses show a clear improvement after therapy.

**Figure 9:**
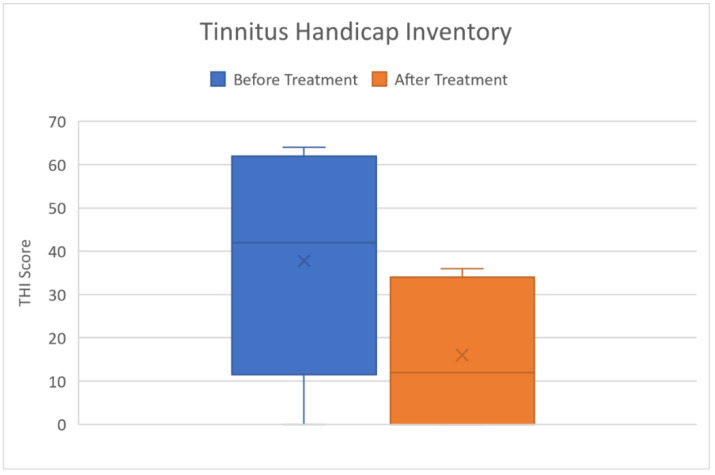
THI showed a significative subjective improvement of Tinnitus after treatment (*p* = 0.0018). Acufenometry values confirmed this trend.

All our patients reported the contextual onset of tinnitus except patient no.2. All had a significant improvement of tinnitus after therapy (Figure 9).

**Figure 10:**
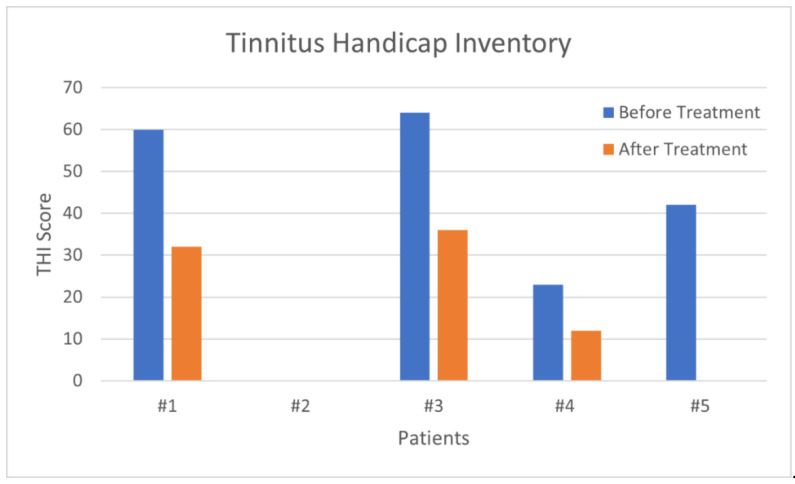
Each patient had an improved THI score after treatment; patient No. 2 was the only one to not experience tinnitus, and patient No.5 fully recovered from tinnitus.

The data relating to each patient’s THI before and after therapy are shown in Figure 10.

Patient no.1 had a complete audiovestibular involvement with HL, tinnitus and vertigo.

All our patients received specific medical treatment for SSNHL including oral high dose steroids, mesoglycan and, once they tested negative for SARS-CoV2, underwent HBOT.

The Oxygen Chamber treatment was a prohibited area for COVID-19 patients. This caused slightly delayed treatment in patients no.2 and no.4: they started the first cycle of treatment 17 days and 11 days after the onset of audiological symptoms, respectively. This could be a major weak point of the therapeutic pathway.

All our patients except patient no.1 received a short-term oral azithromycin treatment. Given that its ototoxic risk is mostly related to high dose and treatment duration, and that SSNHL is often progressive, recent literature has not considered short term azithromycin therapy to be a risk factor for SNHL [57,58,59]. For this reason, we excluded the role of this macrolide in the genesis of our SSNHL cases.

## 5. Conclusions

SSNHL could be an occasional symptom of COVID-19, even in mild manifestations of the disease. Our experience leads us to underline the value of promptly recognizing and addressing uncommon symptoms, giving patients the opportunity to receive early treatment.

For this purpose, we strongly suggest providing knowledge to all healthcare personnel that could be involved in a possible SSNHL assessment, as early recognition is mandatory.

SSNHL is one of the audiological emergencies that needs a diagnostic and therapeutic pathway which should not be barred to COVID-19 patients. We observed that this restriction could be the cause of partial or poor recovery in our patients.

There is a growing body of literature about SSNHL and COVID-19, suggesting the need to aggregate and share experiences about this possible relationship. Further studies on larger samples are needed to give a stronger statistical value to this hypothesis.

## Data Availability

The data presented in this study are available on request from the corresponding author. The data are not publicly available due to privacy restrictions.
